# A Comprehensive Overview on Chemotherapy-Induced Cardiotoxicity: Insights into the Underlying Inflammatory and Oxidative Mechanisms

**DOI:** 10.1007/s10557-024-07574-0

**Published:** 2024-03-16

**Authors:** András Nagy, Denise Börzsei, Alexandra Hoffmann, Szilvia Török, Médea Veszelka, Nikoletta Almási, Csaba Varga, Renáta Szabó

**Affiliations:** Department of Physiology, Anatomy, and Neuroscience, Faculty of Science and Informatics, University of Szeged, Közép Fasor 52, 6726 Szeged, Hungary

**Keywords:** Chemotherapy, Cardiotoxicity, Inflammation, Oxidative stress

## Abstract

While oncotherapy has made rapid progress in recent years, side effects of anti-cancer drugs and treatments have also come to the fore. These side effects include cardiotoxicity, which can cause irreversible cardiac damages with long-term morbidity and mortality. Despite the continuous in-depth research on anti-cancer drugs, an improved knowledge of the underlying mechanisms of cardiotoxicity are necessary for early detection and management of cardiac risk. Although most reviews focus on the cardiotoxic effect of a specific individual chemotherapeutic agent, the aim of our review is to provide comprehensive insight into various agents that induced cardiotoxicity and their underlying mechanisms. Characterization of these mechanisms are underpinned by research on animal models and clinical studies. In order to gain insight into these complex mechanisms, we emphasize the role of inflammatory processes and oxidative stress on chemotherapy-induced cardiac changes. A better understanding and identification of the interplay between chemotherapy and inflammatory/oxidative processes hold some promise to prevent or at least mitigate cardiotoxicity-associated morbidity and mortality among cancer survivors.

## Background

Cardiovascular diseases (CVDs) and cancer are two leading causes of death worldwide. According to the World Health Organization survey in 2020, cancer accounted for nearly 10 *million* deaths (nearly one in six deaths) [1], whereas CVDs claimed an estimated 17.9 *million* lives in 2019 [2]. Although CVDs and cancer possess several overlapping risk factors, it is also important to note that the cardiovascular complications of cancer or cancer therapy can significantly increase the global burden of CVDs [3].

Over the past decades, novel medical therapies in oncology have significantly improved life expectancy among patients with malignancies; however, a growing number of cancer survivors face long-term consequences of cancer therapies with a wide range of unintended side effects. Among chemotherapy-induced adverse effects, cardiotoxicity is one of the biggest issues, affecting the heart [4]. Clinically, cardiotoxicity can be manifested by left ventricular failure, myocardial ischemia, arrhythmias, myocardial infarction, and endothelial dysfunction [5, 6]. Although different types of chemotherapeutic agents are administered in different clinical conditions, duration, and doses, there is increasing evidence that many chemotherapeutic drugs share cardiovascular side effects. Despite extensive research, the underlying mechanisms of chemotherapy-induced cardiotoxicity are not fully elucidated, though it is likely to be multifactorial, in which the inflammatory processes and oxidative damage play an important role [7, 8]. Cytokines (e.g., interleukin-6 (IL-6), IL-1β, tumor necrosis factor-alpha (TNF-α)) that are released during acute and chronic inflammation can influence the function and expression of cardiac ion channels as well as lead to cardiac remodeling and impaired cardiac function [9–11]. In addition to functional and morphological changes in the cardiac tissue, a strong relationship can be associated among cardiovascular disorders, inflammation, and oxidative damage. In a vicious cycle, cardiomyocyte damage can amplify the inflammatory cascade and the accumulation of reactive oxygen and nitrogen species, which worsen the cardiovascular homeostasis and function [12].

Oxidative stress and inflammation are two of the most commonly encountered phenomenons of chemotherapy-induced cardiotoxicity, and both are characterized by a variety of molecular alterations in cardiac cells [13]. Oxidative stress is associated with an increase of reactive oxygen species (ROS) generation (HO•, HO-, O2•-, H2O2, NO•), mitochondrial dysfunction, decrease in antioxidant levels and antioxidant system activity (glutathione (GSH), superoxide dismutase (SOD), catalase (CAT), and glutathione peroxidase (GPx)), and an increase in lipid peroxidation [14]. Furthermore, high levels of intracellular ROS are known to modulate the normal functioning of several signaling pathways, including B-cell lymphoma 2 (Bcl-2), caspase-3, nuclear factor kappa-light-chain-enhancer of activated B cells (NF-κB), protein kinase C (PKC), mitogen-activated protein kinase (MAPK), phosphoinositide 3-kinase/protein kinase B (PI3K/Akt), p38, and c-Jun N-terminal kinase (JNK) [15]. In particular, inhibition of the anti-apoptotic Bcl-2 and overexpression of caspase-3 lead to the apoptotic cell death of cardiomyocytes [16]. Consequently, cell death and shifts in oxidant/antioxidant homeostasis play a key role in the stimulation of inflammatory responses and create an inflammatory microenvironment in the heart. NF-κB is a major pathway in chemotherapy-induced cardiotoxicity via the activation of TNF-α which induces inflammatory reactions through increased levels of interleukins (IL-6, IL-8, IL-1β) [17]. In addition, NF-κB is a transcription factor that increases the synthesis of other proinflammatory molecules, such as cyclooxigenase-2 (COX-2), lipoxigenase-2 (LOX-2), cell-adhesion molecules, chemokines, and inducible nitric oxide synthase (iNOS) [18, 19], subsequently leading to further inflammation-mediated cell death and cardiac damage [20]. These aforementioned oxidative stress and inflammatory parameters are all linked to different types of CVDs, including heart failure, left ventricular dysfunction, and coronary heart disease [21, 22].

Based on these epidemiological data and implications, a better understanding of the molecular and biochemical mechanisms underlying chemotherapy-induced cardiotoxicity provides potential targets for early diagnosis and treatments for patients with cancer. Although most reviews focus on the cardiac changes induced by a selected chemotherapeutic agent [23–26], our aim was to provide a comprehensive review, in which we present cardiotoxic effects caused by various chemotherapeutic agents, including anthracyclines, antimetabolites, alkylating agents, microtubule inhibitors (MTIs), and tyrosine kinase inhibitors (TKIs). Additionally, we focus on the relationship of cardiotoxicity and inflammatory processes as well as oxidative markers, which together can influence the life expectancy of patients with malignancies or the success of cancer therapies.

## Classification of Chemotherapeutic Agents Causing Cardiotoxicity

### Anthracyclines

Anthracyclines are a group of cytotoxic antibiotics with a tetracyclic aglycone base and a sugar moiety attached to the C-7 carbon atom of ring A [27]. They are often used during the treatment of a wide variety of tumors, such as acute lympho- and myeloblastic leukemia; acute lymphocytic and non-lymphocytic leukemia; breast, ovarian, thyroid, gastric, and bronchogenic carcinoma; bone and soft tissue sarcoma; and neuroblastoma. The first anthracycline drug, daunorubicin, an antibiotic pigment synthesized by *Streptomyces peucetius* has been early on associated with antitumor activity [28]. Later, during the 60 s and 70 s, several derivatives of daunorubicin (the most commonly used doxorubicin (DOX), epirubicin, idarubicin, and pirarubicin) were studied and approved for clinical use. In the meantime, it became clear that despite the high efficacy of anthracycline-based chemotherapy, these drugs have a major drawback: cardiotoxicity, a side effect being subject of countless studies, and one that causes concerns to this day. There are several approaches to develop new non-cardiotoxic anthracyclines (aldoxorubicin, DTS-201, camsirubicin, annamycin); however, these are still under rigorous investigation in clinical trials [29].

Anthracyclines have two main mechanisms of action through which they are capable of exerting cytotoxic effects. One of them is their ability to disrupt DNA replication by inhibiting the topoisomerase (TOP2) enzyme [30]. The anthraquinone part of anthracyclines acts as a DNA intercalating domain, and it promotes the stabilization of TOP2-DNA complexes. This leads to an increased number of double-strand DNA breaks, perpetual damaging of the nucleic acid, inhibition of cell proliferation, and cell death [31]. It is important to mention here that there are two isoforms of human TOP2: topoisomerase II-α (TOP2-α) and topoisomerase II-β (TOP2-β). TOP2-α shows an overexpression in proliferating cells, while TOP2-β is found in quiescent cells [32]. This means that the antitumoral effect of anthracyclines in regard to DNA replication and damage comes from targeting the overly active TOP2-α enzymes in cancer cells. However, the effects of anthracyclines are not exclusive to TOP2-α [33]. In tissues like the myocardium, these drugs can start to bind to TOP2-β. This suppresses the peroxisome proliferator-activated receptors and leads to the activation of p53 signaling, disruption of Ca^2+^ homeostasis, and mitochondrial dysfunction, finally causing increased apoptosis in cardiac cells [32].

The other aspect of anthracycline cytotoxicity comes from their capability to significantly increase intracellular ROS generation and activate inflammatory responses linked to NF-κB and TNF-α, to increase IL-1β and IL-6 concentration and to induce apoptosis [12]. ROS generation specifically is thought to be the main mechanism of action for anthracycline-mediated cardiotoxicity, and it is linked to mitochondrial dysfunction and redox cycling [34]. Cardiac cells possess a large number of mitochondria due to their high energy demand, which makes them specifically vulnerable against drugs like DOX which has a high affinity to cardiolipin, leading to mitochondrial DOX accumulation [35]. After DOX binds to cardiolipin, it can disrupt the electron transfer chain (ETC) through the inhibition of complex I, II, and IV. Several oxidoreductases, such as cytochrome P450 reductase and NADP oxidase (NOX), are able to reduce the quinone structure within DOX, forming semiquinone intermediates. These intermediates then convert oxygen into superoxide and indirectly contribute to the increase of hydrogen peroxide and hydroxyl radical levels [36, 37]. Moreover, the presence of these oxidative stressors is directly connected to mitochondrial protein oxidation, lipid peroxidation, ferroptosis, and DNA damage which can lead to further cell death and myocardial contraction impairment [38]. Beside the direct effects on oxidative parameters, anthracyclines also increase the binding activity of NF-κB [39], upregulate the expression of death receptors like tumor necrosis factor receptor 1 (TNFR1), toll-like receptor 4 (TLR4), tumor necrosis factor receptor superfamily member 6 (FasR), death receptor 4/5 (DR4/5), and activate TNF-related apoptosis inducing ligand (TRAIL) signaling, which all lead to cardiomyocyte apoptosis [40, 41].

In vitro studies, focused on the effects of chemotherapy on cardiac cells, show strong correlations between the use of anthracyclines and the dysregulation of the intracellular antioxidant system. A 5-day treatment with 0.2 µM DOX/day has been shown to significantly increase mitochondrial depolarization and DNA fragmentation, as well as ROS and malondialdehyde (MDA) concentrations, and subsequently decrease SOD and CAT activity and GSH content in embryonic ventricular rat heart derived H9c2 cardiomyoblasts [42]. Similar to DOX-induced alterations, Zhang et al. also demonstrated that a 24-h treatment with idarubicin ranging between 1 and 9 µM increases intracellular ROS and MDA concentrations, significantly reduces SOD, CAT, and GSH activity; upregulates nitric oxide synthase (NOS) expression; increases lactate dehydrogenase (LDH) and apoptosis-associated protein levels; and reduces cell viability of the adult murine cardiac cell line HL-1 [43].

Animal studies, conducted mainly on Wistar rats, have also provided valuable insight into the role of oxidative stress and inflammation, through the changes in concentration and activity of proinflammatory cytokines and oxidative parameters. At a cumulative DOX dose of 15 mg/kg [44] observed significant increase in cardiac troponin I, TNF-α, IL-1β, and caspase-3 concentration, as well as decreased total antioxidant capacity and elevated MDA level, with no significant changes in BNP concentration. At the same cumulative dose of 15 mg/kg [45] found that DOX induces significant increase in LDH, CK, and IL-6 levels also, while significantly reducing GSH, GSH-Px, CAT, and SOD activity. According to Al-Kuraishy et al., a single DOX dose of 15 mg/kg also significantly increases cardiac troponin, BNP, caspase-3, and LPO concentration and reduces GSH-Px serum level; however, the elevation showed by MDA and TNF-α concentrations were not significant (*p* > 0.05). Results from research conducted on C57BL/6 J mice show similar changes regarding the antioxidant system and inflammatory processes [46]. At a cumulative DOX dose of 20 mg/kg, Qi et al. demonstrated significant elevation in the concentrations of LDH, CK-MB, troponin T, ANP, BNP, Bcl-2-like protein 4 (BAX), caspase-3, MDA, TNF-α, IL-1β, IL-18, and NF-κB. Moreover, mRNA levels of transforming growth factor-β1 and α-smooth muscle actin (fibrosis markers) and myocardial collagen accumulation were also significantly increased. Meanwhile, expression level of Bcl-2 and GSH to GSSG ratio was both significantly reduced. Interestingly, SOD and GSH-Px activities were slightly increased by the DOX treatment [47]. These changes in molecular concentrations and activities were also accompanied by histologic and physiologic alterations in the myocardium. These included myocardial swelling, myofibrillar disorganization and myofibrillar loss, vacuolization (cytoplasmic and perinuclear), congestion of myocardial vessels, hyalinization, myocytolysis, coagulative necrosis, and areas of interfibrillar edema [44–46] as well as significant decrease in left ventricular ejection fraction (LVEF%) and left ventricular systolic function (LVSF%) [47]. This further strengthens the idea that oxidative stress and inflammation in the myocardium play an important role in the development of anthracycline-induced cardiotoxicity.

**Fig. 1:**
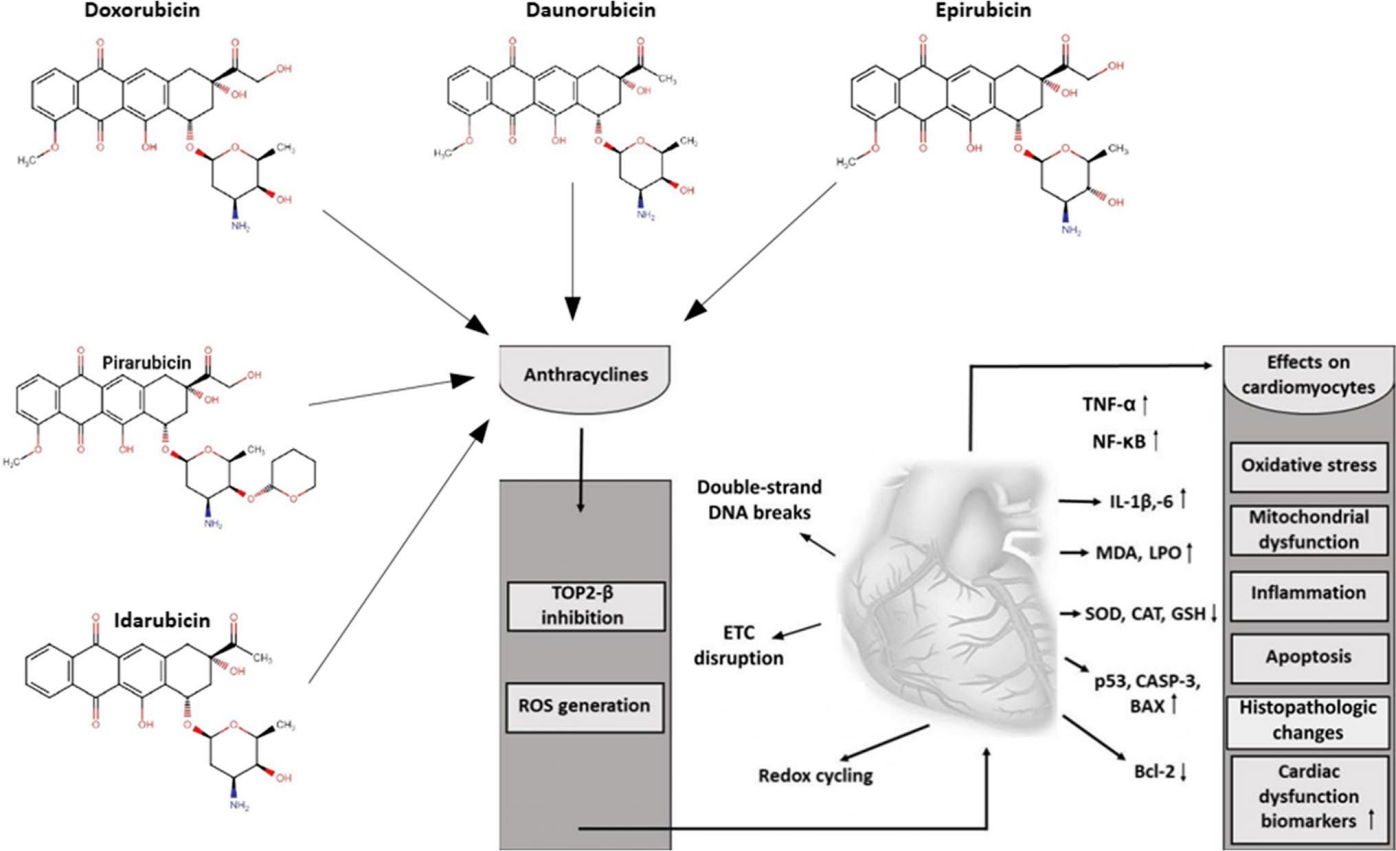
Schematic representation of anthracycline-induced cardiotoxicity. TOP2-β, topoisomerase II-β; ETC, electron transfer chain; TNF-α, tumor necrosis factor-alpha; NF-κB, nuclear factor kappa-light-chain-enhancer of activated B cells; IL-1β,-6, interleukin-1beta,-6; MDA, malondialdehyde; LPO, lipid peroxidation; SOD, superoxide dismutase; CAT, catalase; GSH, glutathione; CASP-3, caspase-3; BAX, Bcl-2-like protein 4; Bcl-2, B-cell lymphoma 2

A growing body of clinical reports also demonstrates that oxidative stress and inflammation-related processes are key mechanisms in DOX-induced cardiotoxicity. According to the results of Skrypnyk et al., signs of changes in cardiac function (shortness of breath during physical activity, palpitations, tachycardia, supraventricular extrasystole, reduced total QRS voltage) after 4–5 days in patients who received DOX treatment were accompanied by a significant increase in lipid peroxidation and contrary to expectations, a (not significant) elevation of SOD concentration. Parameters associated with oxidative stress–induced endothelial damage (increase of NOS concentration and decreased nitrite levels) also showed slight changes; however, these were also not significant [48]. In a clinical study, Trofenciuc et al. underpinned the direct relationship between DOX-related cardiovascular morbidities and TLR4-dependent signaling processes. They found that DOX regimen received for hematological malignancies resulted in a significant increase in TLR4 expression, which can be associated with inflammation, oxidative damages, and apoptosis [49]. In another study, Todorova et al. aimed to determine the correlation among DOX-induced cardiotoxicity, endothelial injury, and plasma inflammatory parameters (such as myeloperoxidase (MPO), C-reactive protein) in patients with breast cancer. In accordance with the previous study, they concluded a strong relationship among the examined parameters [50]. Figure 1 shows a schematic representation of the anthracycline-related mechanisms of action, biomarkers, and its effects on cardiomyocytes.

### Antimetabolites

Antimetabolites are some of the most commonly used chemotherapeutic agents, and they have been integral in cancer treatment since the early 1950s [51]. They are mainly utilized against acute childhood lymphoblastic leukemia, head and neck squamous cell carcinoma, non-Hodgkin’s lymphoma, breast cancer, colorectal cancer, osteosarcoma, and leptomeningeal metastases [52–54].

Interference with DNA synthesis is the main mechanism of action for antimetabolites. Antifolate antimetabolites like methotrexate are capable to competitively inhibit dihydrofolate reductase (DHFR), an enzyme responsible for the conversion of intracellular folates into active dihydrofolate and tetrahydrofolate, thus prohibiting the biosynthesis of nucleotide precursors, leading to cytostasis [55]. However, recent studies have shown that methotrexate is responsible for more than DHFR inhibition. Non-DHFR-mediated effects consist of oxidative stress via increased ROS production [56], induction of cell differentiation [57], DNA demethylation, and protein acetylation [58].

Pyrimidine analogues (cytarabine, 5-fluorouracil (5-FU), capecitabine) have the same cytostatic effect as antifolates, but their mechanism of action is significantly different. In this case, the chemotherapeutic agents are nucleobase analogues (capecitabine and 5-fluorouracil) and nucleoside analogues (cytarabine). 5-Fluorouracil and its prodrug, capecitabine, are both involved in the dysregulation of dTMP synthesis. After intracellular synthesis or entry into the cell, 5-FU is transformed first into 5-fluoro-2′-deoxyuridine (FdURD) by thymidine phosphorylase (TP) and then into fluorodeoxyuridylate (FdUMP) via thymidine kinase (TK). Finally, FdUMP is capable of binding to the nucleotide-binding site of thymidylate synthase (TS), thus inhibiting its capability to transform dUMP into dTMP and prohibiting DNA synthesis [52, 59, 60]. The previously mentioned antifolates are capable of inhibiting TS as well, after they connect to the folate-binding site [61]. Cytarabine also prohibits DNA synthesis. It acts as a competitor to deoxycytidine, inhibiting DNA-polymerase activity, directly preventing DNA replication [62].

The risk of developing cardiotoxicity due to 5-FU and capecitabine treatment is quite significant, the incidence ranging between 0.55 and 19% [63]. The exact mechanism of fluoropyrimidine cardiotoxicity is not fully elucidated. However, several models have been proposed throughout the years that intend to resolve this problem. Common manifestation of cardiotoxicity associated with fluoropyrimidines include atypical chest pain, angina, myocardial infarction, arrhythmias, cardiac inflammation, and heart failure, and the two main propositions for 5-FU and capecitabine-induced cardiotoxicity are ischemia and direct myocardial cell damage [23]. Ischemia in particular is believed to be caused by coronary vasospasm [64, 65], which can be related to endothelial dysfunction and primary smooth muscle dysfunction [23]. Decreased NO release, increased blood viscosity, platelet aggregation, and endothelin-1 all contribute to reduced oxygen carrying capacity in the heart, thus leading to 5-FU-induced myocardial ischemia [66]. In addition, increased ROS levels and decreased antioxidant capacity are associated with direct cardiomyocyte injury, leading to increased oxidative stress and finally causing myocardial inflammation and cardiac myocyte apoptosis [66]. Moreover, fluoroacetate, the byproduct of 5-FU catabolism, can interfere with the Krebs cycle, resulting in increased intracellular fluorocitrate level and alteration with the normal cardiac function [67]. The underlying mechanisms for methotrexate-induced cardiotoxicity are the inhibition of the antioxidant systems and the prohibition of DNA and RNA synthesis which directly affect not only tumors, but cardiac cells as well, resulting in cardiac damage [68, 69].

In a study conducted on H9c2 cardiomyocytes, Dogan et al. [70] demonstrated that treatment MTX treatment ranging between 0.156 and 10 µL significantly decreases cell viability after 48 h. In addition, they also found that MTX significantly increases oxidative stress parameters, such as hypoxia-inducible factor-1α (HIF-1α), advanced oxidation protein products (AOPPs), MDA, lipid hydroperoxide (LOOH), and xanthine oxidase activity (XO), while plasma total thiol (T-SH) concentration, as well as CAT activity and TAC, was also decreased.

**Fig. 2:**
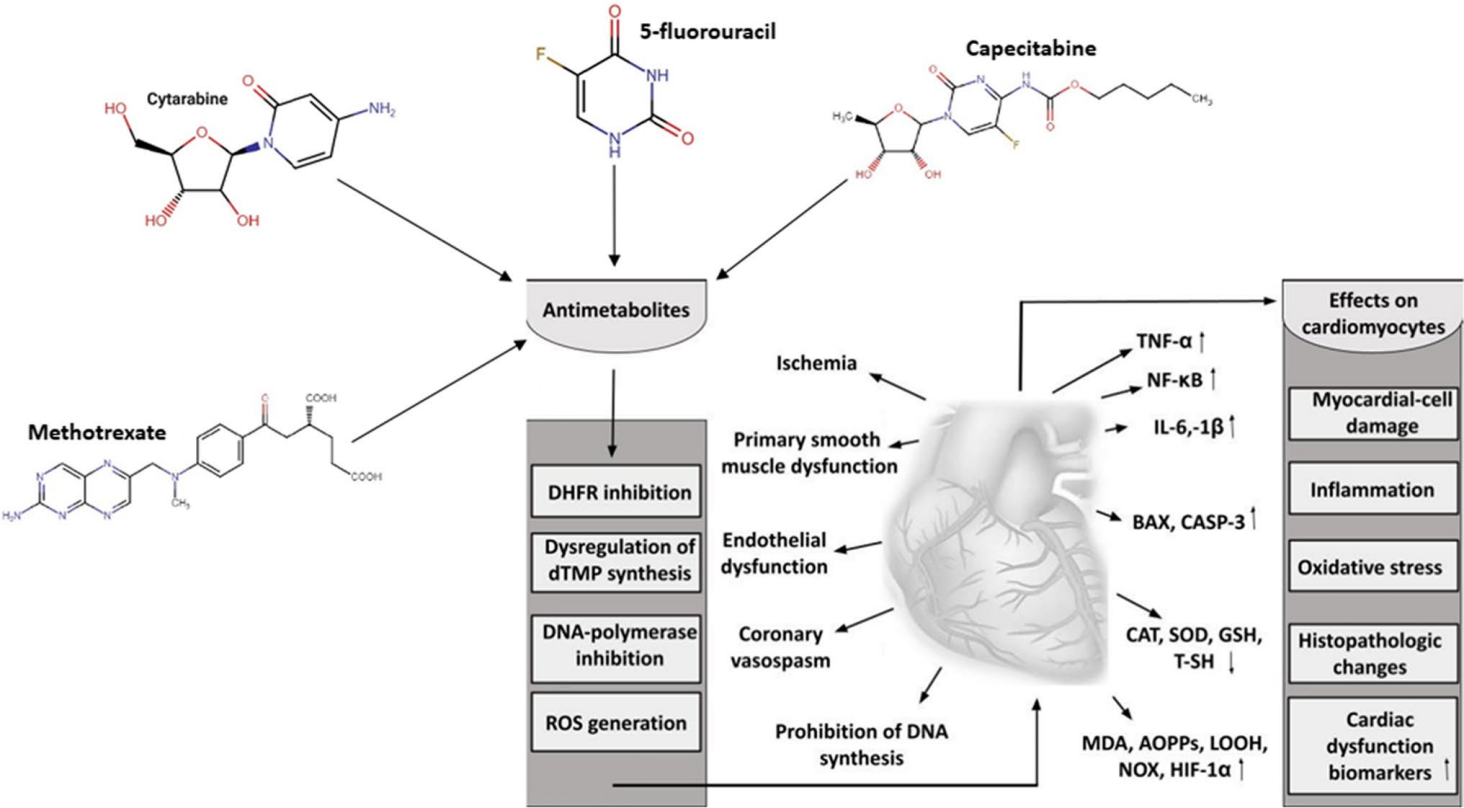
Schematic representation of antimetabolite-induced cardiotoxicity. DHFR, dihydrofolate reductase; TNF-α, tumor necrosis factor-alpha; NF-κB, nuclear factor kappa-light-chain-enhancer of activated B cells; IL-1β,-6, interleukin-1beta,-6; MDA, malondialdehyde; SOD, superoxide dismutase; CAT, catalase; GSH, glutathione; T-SH, total thiol; CASP-3, caspase-3; BAX, Bcl-2-like protein 4; AOPP, advanced oxidation protein products; LOOH, lipid hydroperoxide; NOX, NADP oxidase; HIF-1α, hypoxia-inducible factor-1α

Animal studies focused on pyrimidine analogues also support the previously mentioned findings. 5-FU treatment, with a cumulative dose of 300 mg/kg (weekly intraperitoneal injections for 6 weeks) was able to induce several ECG changes in male Wistar rats, including elevation in the S-T segment, prolonged QTc duration, a 13% drop in heart rate, and a 15% prolongation in the R-R interval [71]. These ECG changes were accompanied by histologic alterations: vacuolization in the sarcoplasmic tissue, intermuscular edema, congestion of myocardial blood vessels, and focal necrosis of cardiomyocytes associated with inflammatory cell infiltration. At molecular levels, significantly elevated contents of cardiac injury biomarkers, such as cardiac troponin I, NT-proBNP, endothelin-1, and thromboxane A2, were present. Oxidative parameters also showed significant change, with increased levels of cardiac NOX, COX-2, and MDA and decreased concentration of GSH. Additionally, 5-FU also demonstrated the capability to activate several biochemical pathways related to apoptosis, inflammation, and oxidative stress, including NF-κB, *p*ERK1/2, caspase-3, Rho-associated protein kinase, AKT, and eNOS [71]. Even at a lower 5-FU dose, oxidative and inflammatory parameters are still significantly altered. Arafah et al. demonstrated that a single injection of 150 mg/kg 5-FU significantly increases LPO, H_2_O_2_, NF-κB, TNF-α, and IL-6/-10/-1β and reduces SOD, CAT, and GSH content. They also found that 5-FU significantly elevated MPO, XO, NO, LDH, MCP, CK-MB, cTn-1, BAX, and caspase-3 levels and caused myocytic degeneration [72]. The same results were obtained by Ibrahim et al. using capecitabine. They found that, at a dose of 140 mg/kg vascular congestions, endothelial hypertrophy, interstitial hemorrhages and edema, Zenker’s degeneration, and microvacuolation of cardiomyocytes appeared in the cardiac tissue. It also reduced serum and cardiac TAC, GSH content, and GPx activity, while increasing MDA, CK-MB, LDH, TNF-α, and IL-1β concentrations and upregulated the mRNA expression of NF-κB and TLR-4 [73]. Figure 2 summarizes the antimetabolite agent-induced mechanisms of action, biomarkers, and their effects on cardiomyocytes.

### Alkylating Agents

The biological effects of alkylating agents were first described at the end of the nineteenth century, and later on during World War I, a subgroup, known as sulfur mustards, was used in chemical warfare. However, during the 1930s and 1940s, a series of research showed potential for nitrogen mustards (cyclophosphamide, ifosfamide) in chemotherapy [74]. In 1969, another group of alkylating-like drugs were shown to be effective anticarcinogens in animal studies. These were the platinum analogues, with cisplatin in particular being authorized for the treatment of ovarian and testicular cancer [75]. Nowadays, these nitrogen mustards and platinum analogues are used for the treatment of lymphomas, multiple myeloma, neuroblastoma, small cell lung carcinoma, ovary and breast adenocarcinoma, malignant testicular germ cell tumors, sarcomas, and cervical and bladder cancer [76–79]. It is also important to mention that their usage as chemotherapeutics has become more limited after a substantial amount of research started to suggest that they are often responsible for uro-, nephro-, and cardiotoxicity [80].

The antitumoral effect of alkylating and alkylating-like compounds comes from their ability to modify DNA, RNA, and protein structures, but their mechanism of action is different in some aspects. Both cyclophosphamide and ifosfamide have to go through a hepatic activation process via the cytochrome P450 enzyme, followed by the transformation into aldophosphamide and aldoiphosphamide, and finally producing phosphoramide mustard and acrolein. Phosphoramide mustard is responsible for DNA cross-linking by alkylating the N7 position of guanine, thus inhibiting DNA separation, replication, and repair. Acrolein is also believed to cause DNA damage, but some findings also suggest that it is responsible for severe glutathione (GSH) depletion, leading to apoptosis [81]. Cisplatin works somewhat similarly to phosphoramide: it is capable of binding to the N7 position of guanine. However, unlike phosphoramide mustard, cisplatin is not alkylating guanine, but creates a covalent bond via its platinum atom [82]. Moreover, cisplatin also causes RNA cross-linking and DNA-polymerase inactivation and alters the function of several other proteins (Na^+^/H^+^ exchanger protein, tubulin, and thioredoxin reductase) [83].

Cyclophosphamide and ifosfamide-induced cardiotoxicity is a well-known and documented side effect in oncology. The risk of heart failure is estimated to increase between 7 and 28% following cyclophosphamide treatment [84] and 17 to 30% following ifosfamide treatment [85]. However, the exact mechanism for nitrogen mustard-induced cardiotoxicity is not fully understood yet; multiple theories have been proposed over the years. One leading hypothesis in this area is that the intermediate metabolites can induce the generation of ROS, leading to mitochondrial and endothelial capillary damage, followed by ischemic myocardial injury, arrhythmias, hypertension, thromboembolisms, and pericarditis [26]. The findings of Kurauchi et al. also confirm the plausibility of this theory. They found that exposure to cyclophosphamide metabolites (4-hydroxy-cyclophosphamide and acrolein) caused ROS accumulation and myocardial toxicity in the rat cardiac myocardial cell line H9c2 [86]. Cisplatin is similar in this aspect, causing increased oxidative stress, mitochondrial abnormalities, left ventricular dysfunction, and myocardial infarction even 20 years after the chemotherapeutic treatment [87]. Altena et al. observed that tissue velocity imaging of early diastole (TVI Et) increased from a baseline of 0 to 4.5% in 10 months and to 16.7% after 6.9 years after the cisplatin treatment [88].

Cyclophosphamide and cisplatin are the alkylating agents most associated with oxidative stress and inflammation affecting the heart. According to Dugbartey et al., cisplatin damages nuclear and mitochondrial DNA, increases ROS production and lipid peroxidation, reduces glutathione and SOD levels, induces mitochondrial membrane depolarization and mitochondrial ultrastructural abnormalities, and activates the transcription of pro-apoptotic genes [89]. In a similar manner, cyclophosphamide also induces ROS generation; enhances NF-κB phosphorylation; increases expression of COX-2, TNF-α, and IL-1β; and activates p38 and p53, leading to oxidative injury, apoptosis, cardiomyopathy, myocardial hypertrophy, and heart failure [26]. Qian et al. demonstrated the ROS-mediated apoptotic effect of cisplatin on H9c2 cells. They found that 1–40 µL cisplatin significantly decreased cell viability by increasing ROS levels and caspase-3, caspase-8, and caspase-9 concentration, activating ERK1/2 pathway and depolarizing the mitochondrial membrane [90]. Cyclophosphamide shows similar results.

**Fig. 3:**
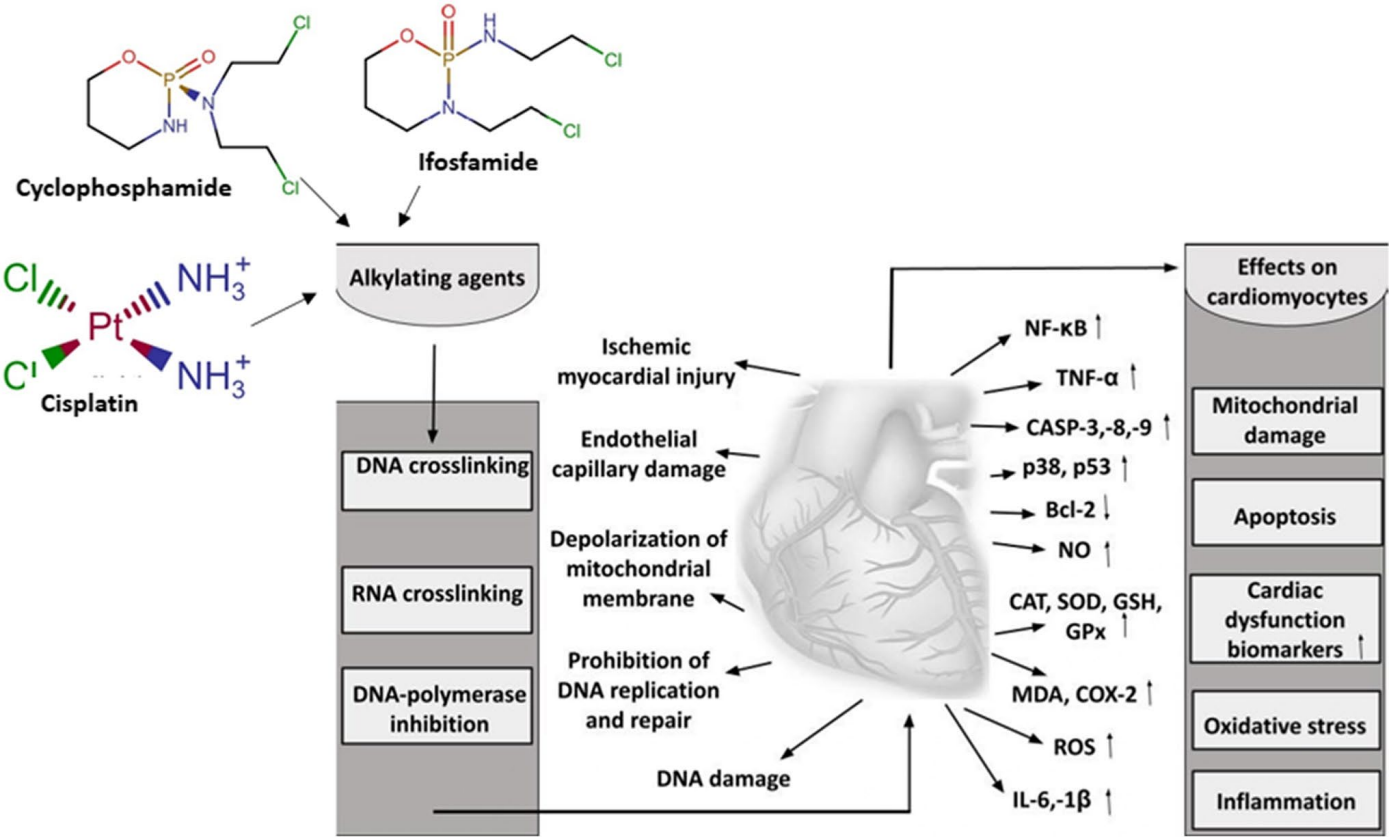
Schematic representation of alkylating agents-induced cardiotoxicity. TNF-α, tumor necrosis factor-alpha; NF-κB, nuclear factor kappa-light-chain-enhancer of activated B cells; IL-1β,-6, interleukin-1beta,-6; MDA, malondialdehyde; SOD, superoxide dismutase; CAT, catalase; GSH, glutathione; CASP-3,-8,-9, caspase-3,8,9; Bcl-2, B-cell lymphoma 2; NO, nitric oxide; ROS, reactive oxygen species; COX-2, cyclooxigenase-2

Similar results were also confirmed by El-Agamy et al. with experiments involving adult male Wistar rats. They found that a single dose of 200 mg/kg cyclophosphamide increased CK-MB, LDH, and MDA concentration and reduced SOD activity and glutathione content in the heart. It also led to the overexpression of TLR4 and NF-κB, overproduction of TNF-α and nitric oxide (NO), the activation of pro-apoptotic caspase-3 signaling, and the inhibition of anti-apoptotic Bcl-2 signaling [91]. Furthermore, cisplatin was also associated with proinflammatory reactions mediated and oxidative stress. Abdellatief et al. [92] demonstrated that a single dose of 5 mg/kg cisplatin can significantly decrease CAT, SOD, GSH, and GSH-Px activity and increase MDA concentration. Furthermore, inflammatory and cardiac injury biomarkers, such as LDH, CK, CK-MB, TNF-α, and IL-6, also presented significantly increased levels. Additionally, a number of histopathological changes were also observed in the cardiac tissue, including disarrayed cardiac muscle fibers, cardiac hypertrophy, congestion of blood vessels, and interstitial edema. Figure 3 gets insights into the alkylating agent–induced mechanisms of action, molecular biomarkers, and their effects on cardiomyocytes.

### Microtubule Inhibitors (MTIs)

Microtubule inhibitors can be divided in two major groups: taxanes (paclitaxel, docetaxel), produced by the plants belonging to the *Taxus* genus, and vinca alkaloids, extracted from the leaves of *Catharanthus roseus* (vinblastine, vincristine) or semi-synthetically produced analogues (vinorelbine, vindesine, vinflunine) [93]. After paclitaxel received approval from the Food and Drug Administration (FDA) as a treatment method for ovarian cancer in 1992 [94], other MTIs followed soon, and they have become reliable chemotherapeutic options for breast and lung carcinomas, non-small cell cancer, and metastatic prostate cancer [95].

In comparison with other chemotherapeutic agents, MTIs have a relatively simple antitumoral mechanism of action. Both taxanes and the vinca alkaloids alter the polymerization process of microtubules, inhibiting mitosis and leading to apoptosis. Taxanes are capable of stimulating the polymerization of tubulin proteins into microtubules and then fixating their structure in such a way that depolymerization becomes impossible. On the other hand, vinca alkaloids work in the exact opposite manner, by blocking the polymerization process and the genesis of microtubules [96].

Most research suggest that the main risk of taxane or vinca alkaloid use in regard to cardiotoxicity is their synergistic effect during combined therapy with anthracyclines, alkylating agents, and monoclonal antibodies (most notably, DOX, cyclophosphamide, and trastuzumab) [97]. Moreover, Sledge et al. demonstrated that paclitaxel monotherapy had a 3.7% incidence of cardiac complications compared to 8.7% after DOX monotherapy and 8.6% after combined therapy with paclitaxel and DOX [98]. In addition, Mikaelian et al. identified cell cycle arrest in endothelial cells as the primary cardiotoxicity mechanism of tubulin-binding drugs [99]. MTIs had been associated with hypertensive cardiac toxicities [100], heart failure, myocardial ischemia, arrhythmia, and pericardial effusion [101], but consistent evidence is seldomly available, to the point that more research is required in the future to clarify the information regarding this topic.

A few studies do present evidence for connection between taxanes and oxidative stress as well as inflammatory responses in cardiac tissue. According to Lage et al., docetaxel decreased the viability of H9c2 cells in a dose-dependent manner (5 nM–10 µM), increased necrosis and apoptosis, while elevating caspase-3 levels. Interestingly, CAT and GPx showed elevated mRNA levels without significant protein concentration change. Conversely, SOD mRNA level remained unchanged; however, SOD protein content increased more than twofold [102]. Ren et al. presented evidence of apoptotic imbalance in mouse cardiomyocytes treated with paclitaxel [103]. The treatment (1–3 mg/kg) increased serum TNF-α as well as BAX and JNK signaling. Additionally, Ali et al. demonstrated that a cumulative paclitaxel dose of 24 mg/kg results in LDH and CK-MB elevation, increased lipid peroxidation, and reduced cardiac GPx and SOD activity and decreased GSH content [104].

The data regarding vinca alkaloids is rather contradicting. Despite evidence for cardioprotective effects of vincristine pretreatment via the reduction of cytochrome C release into the cytosol and the overall activation of pro-survival signaling pathways [105], newer studies show that chronic treatment with vincristine (100 µg/kg) induces TNF-α-mediated cardiac inflammation and oxidative stress via increased iNOS and eNOS expression [106].

**Fig. 4:**
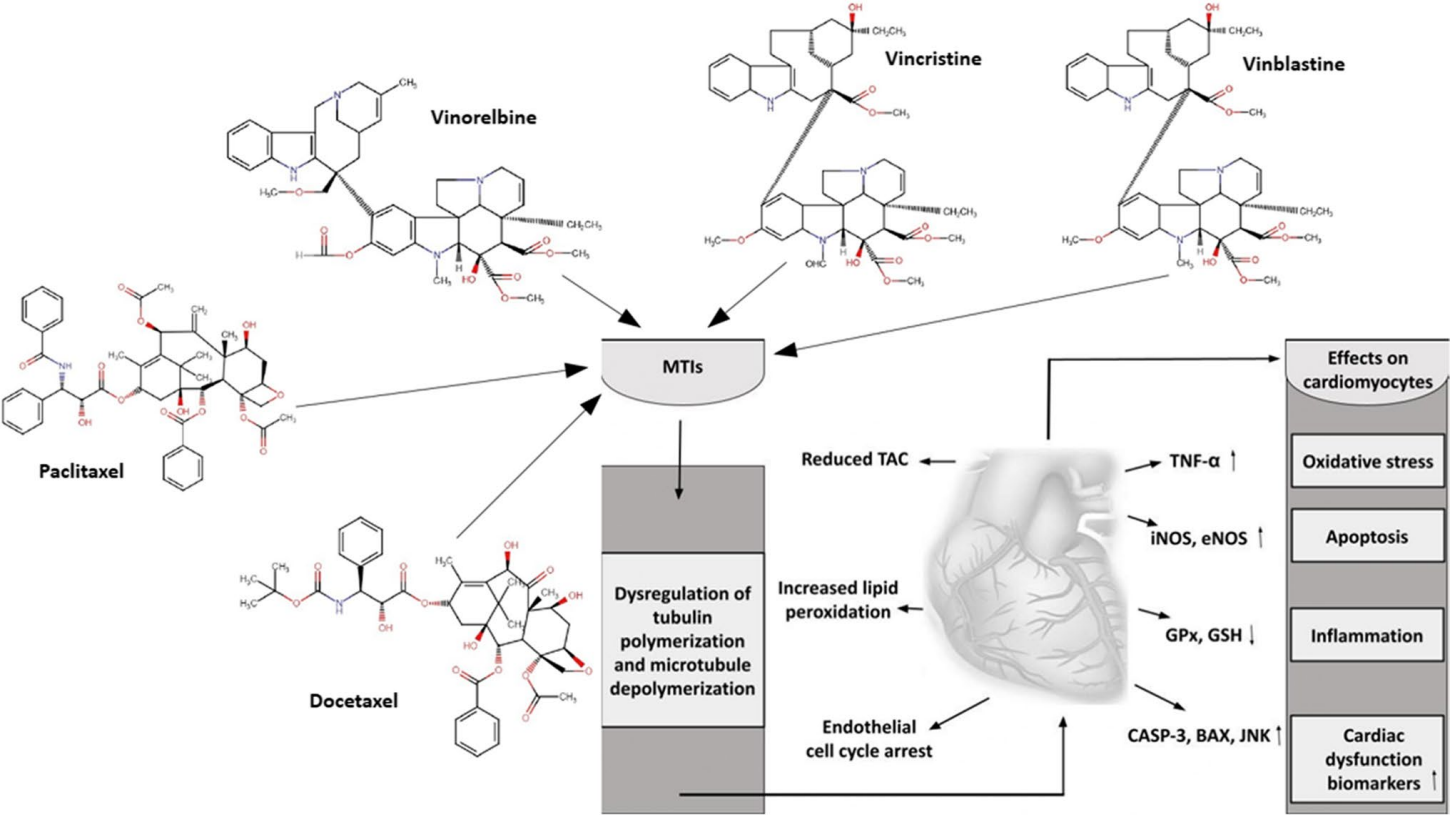
Schematic representation of microtubule inhibitor-induced cardiotoxicity. MTIs, microtubule inhibitors; TAC, total antioxidant capacity; TNF-α, tumor necrosis factor-alpha; iNOS, eNOS, inducible and endothelial nitric oxide synthase; GSH, glutathione; GPx, glutathione peroxidase; CASP-3, caspase-3; BAX, Bcl-2-like protein 4; JNK, c-Jun N-terminal kinase

Moreover, Werida et al. demonstrated in a clinical setting that combined treatment with four cycles of DOX and cyclophosphamide, followed by 12 cycles of paclitaxel, resulted in a significant decrease in left ventricular ejection fraction and a significant increase in TNF-α, MDA, brain natriuretic peptide (BNP), and neurotensin serum levels [107]. Figure 4 summarizes the MTI-induced mechanisms of action, molecular biomarkers, and its effects on cardiomyocytes.

### Tyrosine Kinase Inhibitors (TKIs)

Tyrosine kinase inhibitors are a class of chemotherapeutic agents with the ability to target tyrosine kinase enzymes resulting in the disruption of cellular signaling pathways. Clinical trials for TKI chemotherapy were first conducted during the late 1990s [108], and since then, their use had been approved for the treatment of several types of cancer: chronic myeloid leukemia [109], gastrointestinal stromal tumor [110], metastatic breast cancer [111], metastatic renal cell carcinoma [112], hepatocellular carcinoma [113], HER2-positive breast cancer [114], metastatic colorectal cancer, non-small cell lung cancer, glioblastoma, ovarian cancer, and cervical cancer [115]. They can be separated in two major categories: small molecule TKIs (smTKIs), capable of entering the cell, and monoclonal antibodies (mAbs) that exert their effect on the outer surface of the cell membrane [116]. A few notable examples include imatinib, lapatinib, sunitinib, and sorafenib from the smTKI group and trastuzumab, pertuzumab, and bevacizumab from the mAb group.

Growth factors and their receptors are essential for the signal transduction that activates intracellular molecular pathways and biochemical cascades connected to cell proliferation, differentiation, migration, and apoptosis. Under normal circumstances, the transcription of tyrosine kinases and tyrosine kinase receptors is tightly regulated; however, due to a number of critical mutations, cancer cells lose their ability to downregulate the expression of certain enzymes, causing tumor cells to uncontrollably divide, migrate, induce angiogenesis, and prohibit programmed cell death. The most important proteins in these processes are the vascular endothelial growth factors (VEGF), platelet-derived growth factors (PDGF), epidermal growth factors (EGF), and their receptors (VEGFR, PDGFR, ErbB). By targeting these enzymes, TKIs are capable of disrupting the signal transduction, thus combating the pathological effects caused by the cancerous mutations.

One of the main antitumor mechanisms exerted by TKIs is the prohibition of angiogenesis. Bevacizumab was the first drug developed and approved specifically for anti-angiogenic cancer treatment [117]. It is capable of inhibiting VEGF and subsequently significantly reducing vascularization in the tumor environment [118]. In a similar way, sorafenib and sunitinib are able to inhibit VEGFR and PDGFR activity by competing with ATP at its binding site, leading to a lack of protein phosphorylation and resulting in significant reduction in cancer induced angiogenesis [119, 120]. Imatinib, one of the first FDA-approved TKI drugs, is capable of targeting PDGFR, but it also inhibits the function of BCR-ABL tyrosine kinase, an enzyme responsible for preventing programmed cell death [121]. Lapatinib on the other hand targets two enzymes from the ErbB family of proteins: EGFR and HER2. By inhibiting their activity, lapatinib directly downregulates the Ras-Raf-MEK-MAPK and PI3K/AKT pathways, prohibiting increased cell proliferation and the inhibition of apoptosis [122]. On the same note, trastuzumab and pertuzumab are also capable of binding to ErbB2 (or HER2), preventing its activation through both homo- and heterodimerization [123]. It is also important to mention that there are some arguments against the definition of mAbs as TKIs because their mechanism of action differs from smTKIs [124], but for the scope of this review, we will regard them as such.

TKI-induced cardiotoxicity is well documented, but the exact mechanism of action in some cases is not yet fully understood. Trastuzumab-induced cardiotoxicity (TIC) is the most problematic side effect of targeted HER2-positive breast cancer. The most plausible theory so far, regarding the molecular mechanism of TIC, is based on the fact that the NRG1/HER signaling pathway plays a significant role in maintaining normal cardiac function, thereby trastuzumab, which binds to HER2 and prevents HER2-HER4 heterodimerization, directly alters the MAPK and PI3K/Akt pathways [125]. The disruption of these metabolic pathways is believed to induce ROS production, sarcomere disruption, and myofibrillar structure destabilization, leading to cardiotoxicity [126]. Results from early clinical trials show an incidence of cardiac dysfunction between 3–7% according to the criteria of the Cardiac Review and Evaluation Committee and 2–4% according to the criteria of the New York Heart Association, in patients who underwent trastuzumab monotherapy [127]. However, both trastuzumab + anthracyclines and trastuzumab + paclitaxel combined treatments caused higher rates of cardiac dysfunction (43% and 15% respectively), while trastuzumab + cisplatin treatment had a cardiac dysfunction incidence of 6%. More recent clinical trials and reviews support the observations of the incidence of TKI-mediated cardiovascular events being lower than 10% [128] and suggest that trastuzumab-induced cardiotoxicity is characterized by ventricular dysfunction, reversible myocardial inflammation and edema [129], Q-T prolongation, arrhythmia, and hypertension [130]. Conversely, the use of pertuzumab treatment comes with a more restrained effect regarding cardiac dysfunction. Despite a slight increase in the risk of heart failure, according to Alhussein et al., this was not associated with left ventricular systolic dysfunction, and the authors would generally not recommend against the use of pertuzumab treatment patients with low cardiac risk [98]. Regarding smTKI, the variety of molecular mechanisms related to cardiotoxicity is just as vast. Mitochondrial impairment and oxidative stress are likely to be the basis of cardiomyocyte cell death after imatinib treatment [131], while sunitinib-induced cardiotoxicity seems to be heavily related to the autophagic degradation of cellular communication network 2 factor (CCN2) [132]. Sorafenib represents a special case in this category, because not only does it cause myocyte cell death through necrosis, but it also induces stem cell apoptosis, preventing the generation of new cardiac myocytes leading to even more cardiac damage [133]. This is also in accordance with the findings of Grabowska et al., who reconstructed the cardiomyocyte apoptosis signaling network and using a computational model determined that sorafenib is highly likely to induce cardiotoxicity via apoptosis in cardiomyocytes [134].

Currently, the most accepted theory regarding the role of oxidative stress and inflammatory responses in TKI treatment is that the inhibition of HER2 signaling is highly connected to increased ROS generation and activation of multiple pro-apoptotic pathways [16]. Using human-induced pluripotent stem cell-derived cardiomyocytes and neonatal rat cardiac myocytes, Wang et al. found that afatinib, sorafenib, and ponatinib induced different levels of lipid peroxidation and ROS as well as a significantly increased cardiac troponin T2 content [135]. After 3 h, the percent of cells with high ROS content increased by 14.1%, 16.2%, and 6.8% using 10 µM of afatinib, sorafenib, and ponatinib, respectively. Interestingly, after 24 h, the percent of these cells was lower than at the 3-h mark, indicating a possible transient aspect to TKI-induced ROS production. On the other hand, lipid peroxidation showed a significantly higher increase after 24 h, compared to 3 h. In addition, the expression of proinflammatory genes, such as *Nfkb1*, *Il-6*, *Tnf*, *Txnip*, and *Il1b* were also significantly increased by the three aforementioned drugs [135]. In regard to smTKIs, Bouitbir et al. also demonstrated that 10 µM of sunitinib decreases ΔΨm, reduces GSH concentration, induces H_2_O_2_ production, and activates caspase-3 and caspase-7 signaling, leading to the apoptosis of H9c2 cells [136]. Moreover, combined treatment of pembrolizumab and trastuzumab (200 nM each) significantly decreases the cell viability of human fetal cardiac cells, while activating NF-κB signaling and increasing IL-8 and IL-1β, but not IL-6 [137].

**Fig. 5:**
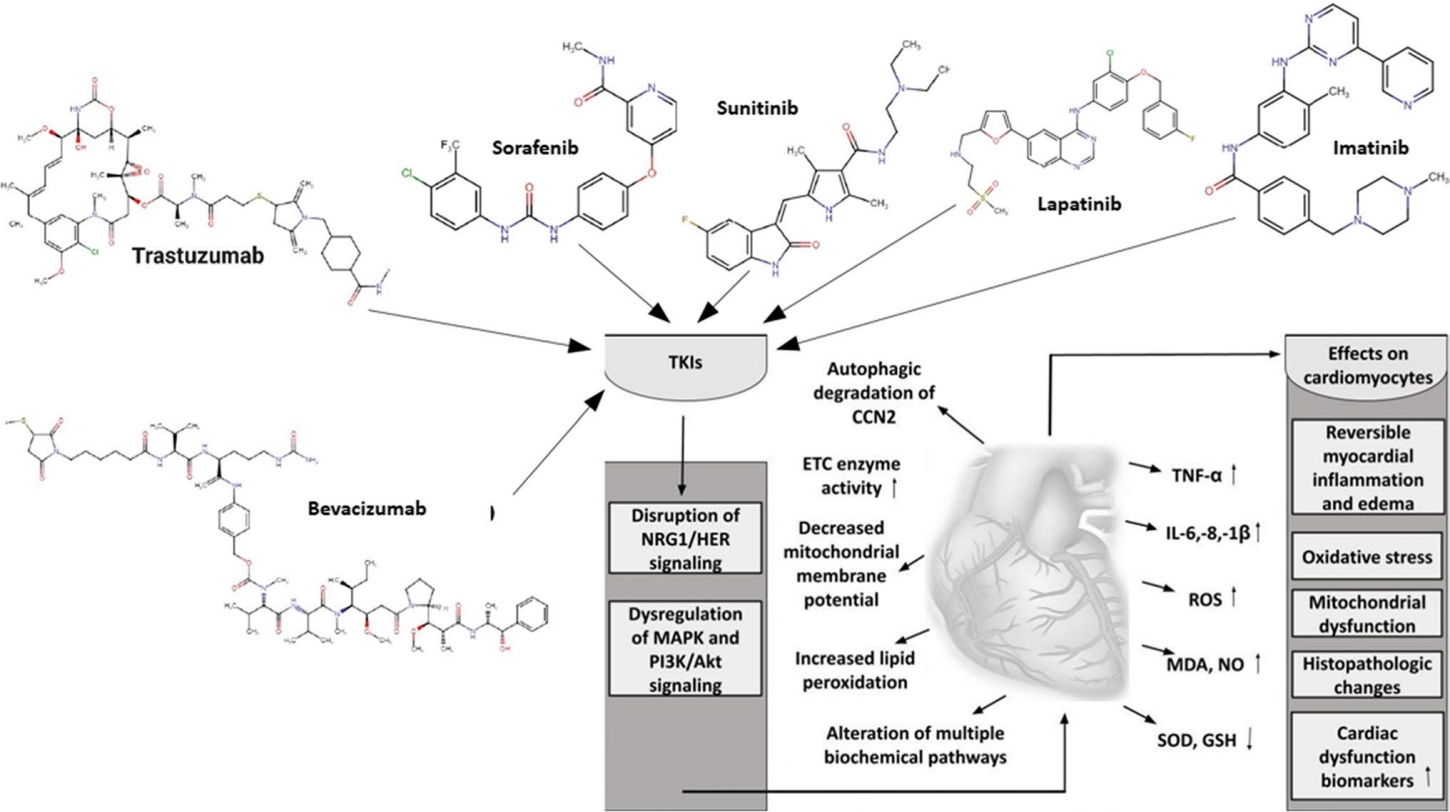
Schematic representation of tyrosine kinase inhibitor-induced cardiotoxicity. TKIs, tyrosine kinase inhibitors; MAPK, mitogen-activated protein kinase; PI3K/Akt, phosphoinositide 3-kinase/protein kinase B; CCN2, cellular communication network 2; ETC, electron transfer chain; TNF-α, tumor necrosis factor-alpha; GSH, glutathione; SOD, superoxide dismutase; NO, nitric oxide; MDA, malondialdehyde; IL-1β,-6,-8, interleukin-1beta,-6,-8; ROS, reactive oxygen species

Beside cell cultures, Bouitbir et al. demonstrated that in mice, a cumulative sunitinib dose of 105 mg/kg (7.5 mg/kg daily, for 2 weeks) increases plasma concentration of troponin I and CK-MB, while decreasing the activity of ETC enzyme complexes and increasing mitochondrial ROS generation and caspase-3 activity [137]. Furthermore, imatinib is also responsible for the induction of oxidative stress and activation of inflammation. Mansour et al. found that at a cumulative dose of 700 mg/kg (100 mg/kg daily for a week), imatinib significantly increases TNF-β and IL-6 concentrations, activates MAPK signaling, decreases SOD and GSH content, and increases MDA and NO concentration and the BAX/Bcl-2 ratio. Additionally, imatinib also caused histologic alterations, characterized by myocarditis, hyalinized myocardial muscles, congested blood vessels, and mononuclear cell infiltration [138]. The summary of TKI-induced cardiotoxicity and its mechanisms and biomarkers are presented in Fig. 5.

## Conclusions

Our comprehensive review summarizes that both oxidative stress and inflammation are significant factors in the development and progression of chemotherapy-induced cardiotoxicity. Their pathogenesis cannot be narrowed down to a single cause; it can be related to multifactorial mechanisms. Right now, there are multiple strategies aiming to reduce the cardiotoxic aspect of chemotherapeutic agents. One strategy that reduces toxicity is the implementation of liposomal delivery systems, which has been adapted for anthracyclines. Myocet®, which consists of a phosphatidylcholine and cholesterol based membrane wrapped around DOX, has been associated with a significantly lower chance to induce cardiotoxicity than non-liposomal forms of treatment, due to lower plasma concentration of free DOX [139]. Drugs like Myocet® and Doxil® that are currently in clinical use present yet another possible solution for the circumvention of cardiotoxicity as a limiting factor in anthracycline therapy [140]. Based on all these, a better understanding of the underlying mechanism and potential targets of the complex inflammatory and oxidative signaling systems might lead to novel approaches in the treatment and prevention of CVDs during and after chemotherapy.

## Data Availability

Not applicable.
